# Comparison of acute versus convalescent stage high-sensitivity C-Reactive protein level in predicting clinical outcome after acute ischemic stroke and impact of erythropoietin

**DOI:** 10.1186/1479-5876-10-6

**Published:** 2012-01-05

**Authors:** Kuo-Ho Yeh, Tzu-Hsien Tsai, Han-Tan Chai, Steve Leu, Sheng-Ying Chung, Sarah Chua, Yung-Lung Chen, Hung-Sheng Lin, Chun-Man Yuen, Hon-Kan Yip

**Affiliations:** 1 Division of cardiology Division of Cardiology, Department of Internal Medicine; Chang Gung Memorial Hospital-Kaohsiung Medical Center, Chang Gung University College of Medicine, Kaohsiung. Taiwan; 2 Center for translational research in biomedical science Chang Gung Memorial Hospital-Kaohsiung Medical Center, Chang Gung University College of Medicine, Kaohsiung. Taiwan; 3 Department of Neurology Chang Gung Memorial Hospital-Kaohsiung Medical Center, Chang Gung University College of Medicine, Kaohsiung. Taiwan; 4 Department of Neurosurgery; Chang Gung Memorial Hospital-Kaohsiung Medical Center, Chang Gung University College of Medicine, Kaohsiung. Taiwan

**Keywords:** acute ischemic stroke, high-sensitivity C-reactive protein, erythropoietin, adverse clinical outcome

## Abstract

**Background and Aim:**

Currently, no data on the optimal time point after acute ischemic stroke (IS) at which high-sensitivity C-reactive protein (hs-CRP) level is most predictive of unfavorable outcome. We tested the hypothesis that hs-CRP levels during both acute (48 h after IS) and convalescent (21 days after IS) phases are equally important in predicting 90-day clinical outcome after acute IS. We further evaluated the impact of erythropoietin (EPO), an anti-inflammatory agent, on level of hs-CRP after acute IS.

**Methods:**

Totally 160 patients were prospectively randomized to receive either EPO therapy (group 1, n = 80) (5,000 IU each time, subcutaneously) at 48 h and 72 h after acute IS, or placebo (group 2, n = 80). Serum level of hs-CRP was determined using ELISA at 48 h and on day 21 after IS and once in 60 healthy volunteers.

**Results:**

Serum level of hs-CRP was substantially higher in all patients with IS than in healthy controls at 48 h and day 21 after IS (all p < 0.001). Levels of hs-CRP did not differ between group 1 and 2 at 48 h and day 21 after IS (all p > 0.5). Multivariate analysis showed that hs-CRP levels (at 48 h and day 21) were independently predictive of 90-day major adverse neurological event (MANE) (defined as recurrent stroke, NIHSS≥8, or death) (all p < 0.03), whereas EPO therapy was independently predictive of reduced 90-day MANE (all p < 0.02).

**Conclusion:**

EPO therapy which was independently predictive of freedom from 90-day MANE did not alter the crucial role of hs-CRP levels measured at 48 h and 21-day in predicting unfavorable clinical outcome after IS.

## Background

Numerous studies have already shown that inflammation plays a crucial role in the initiation of endothelial dysfunction, atherosclerosis and plaque formation, propagation of plaque burden, and finally, rupture of the vulnerable plaque and acute arterial obstructive syndrome (AOS) [1-4]. Although a myriad of inflammatory biomarkers have been reported to be useful in predicting endothelial dysfunction and the likelihood of AOS, high-sensitivity C-reactive protein (hs-CRP) remains one of the most extensively studied and widely accepted inflammatory biomarkers in our daily clinical practice [4-10].

Indeed, not only is hs-CRP an inflammatory biomarker, but it has also been proved to directly participate in the inflammatory process contributing to acute AOS [3,4,7]. Besides, immense clinical observational studies have demonstrated that serum level of hs-CRP is a useful and powerful inflammatory marker in predicting future cardiovascular and cerebrovascular events in patients with and without obstructive arterial disease [3,4,7-11].

CRP, an acute-phase reactant, is synthesized and secreted in the liver 6 h after an acute inflammatory stimulus [12,13] regardless of the etiologies. Both acute myocardial infarction6 and acute ischemic stroke (IS) [14,15] promptly induce an elevation in circulatory hs-CRP from ischemic tissue damage. The half life of hs-CRP in circulation is about 24 hours [16]. Since its relatively short half-life implies that its serum level should return to the baseline level two weeks after an acute ischemic insult such as acute AOS, researchers usually focus on the role of hs-CRP level during acute phase [4,6,14,15] rather than during recovery phase in predicting clinical outcome. On the other hand, whether serum hs-CRP level during acute or convalescent phase is more accurate in predicting clinical outcome in patients after acute IS remains uncertain.

Not only has erythropoietin (EPO) been shown to enhance erythropoiesis under anemic condition, but it has also been reported to be anti-apoptotic [17,18], anti-inflammatory [19,20], and to play a role in the mobilization of endothelial progenitor cells (EPCs) to circulation and angiogenesis [21-23]. Our recent study [24] has demonstrated that EPO therapy significantly improved 90-day clinical outcome in patients after acute IS. However, the study [24] did not show the effect of EPO on the serum level of hs-CRP. Accordingly, this study not only examined whether serum hs-CRP levels during acute and convalescent phases play equally important role in predicting 90-day clinical outcome in patients after acute IS, but it also investigated the impact of EPO therapy on serum hs-CRP level.

## Materials and methods

### Study Design

This study was a sub-study of our recently reported clinical trial [24] which was approved by the Institutional Review Committee on Human Research at Chang Gung Memorial Hospital (No 96-1381A) in 2007 and conducted at Kaohsiung Chang Gung Memorial Hospital. The clinical trial [24] registration number was ISRCTN71371114.

Our recently reported clinical trial [24] was prospective, randomized, and placebo-controlled. The primary end-point was to examine the safety and efficacy of two doses of EPO (Epoetin beta, Roche) (5,000 IU each time, subcutaneous) administered at 48 h and 72 h after acute IS in reducing 90-day combined endpoint of recurrent stroke or death, while the secondary end-point was to assess the serial changes of circulating EPC level in patients after acute IS [24]. Furthermore, the study [24] was designed to evaluate the impact of EPO therapy on the combined adverse neurological event (MANE) [defined as recurrent stroke, National Institutes of Health Stroke Scale (NIHSS) ≥ 8, or death] and on the serum level of hs-CRP after acute IS. Instead of EPO, the placebo-control subjects received 1 mL of subcutaneous normal saline at 48 h and 72 h after acute IS.

### Definitions, Sample Size, Inclusion and Exclusion Criteria

Stroke was defined as a sudden onset of loss of global or focal cerebral function persisting for more than 24 hours [24]. Patients of all ages with acute IS were eligible. Inclusion criteria according to a previous clinical trial [22] included a scoring > 2 on the NIHSS (scores up to 8 indicate moderate neurological status disability) and a time window of ≤ 48 h from onset of symptoms to blood sampling (at 48 h after IS) and study drug administration (time to treatment just after blood sampling). Patients with history of the followings were excluded from the study: intracranial hemorrhage, surgery or trauma within the preceding 3 months, abnormal liver function, renal insufficiency (serum creatinine > 1.5 mg/dL), malignancy, febrile disorders, acute or chronic inflammatory disease at study entry, liver cirrhosis, atrial fibrillation, congestive heart failure, contraindications for Magnetic Resonance Imaging (MRI) examination, no evidence of acute IS on MRI study.

Additionally, patient who had history of allergy to EPO, hematological disorders including myeloproliferative disorder, leukemia, thrombocythemia, polycythemia, past history of deep vein thrombosis, abnormal elevation of hemoglobin (male > 14.5 gm/dL; female > 13.5 gm/dL), or received thrombolytic therapy upon presentation were also excluded from this trial.

An estimated sample size of 157 patients was based on the effective size with an α = 0.05, a power of 80%, a difference in circulating level of EPCs between the patients with NIHSS < 12 and NIHSS ≥12 at 48 h after acute IS of 0.3%, and a standard deviation of 1.0% in patients with NIHSS < 12 and 0.9% in patients with NIHSS ≥ 12 at 48 h after acute IS. A 20% rate of protocol violations was assumed. The calculation of sample size for specific objective was based on our recent report [24].

An overview of the study protocol has been shown in the clinical trial in details [24]. From October 2008 through March 2010, consecutive patients with acute IS were enrolled by the responsible neurologists at the institute. Eighty-three patients constituted the EPO therapy group in the clinical trial [24]. Because three serum samples were missed, the remaining 80 patients were enrolled into the present study as group 1. Additionally, 84 patients constituted the placebo control group in the clinical trial [24]. Also, since four serum samples were lost, the remaining 80 patients were enrolled into the current and assigned as group 2.

Sixty age- and gender-matched healthy volunteers were also studied for circulating level of EPCs and serum level of hs-CRP. Informed consent was obtained from all study subjects.

### Assessment of Neurological Function

Evaluation of severity of neurological impairment in the patients with stroke was based on the National Institutes of Health Stroke Scale (NIHSS) [25] during the acute (at 48 h), convalescent (on day 21), and chronic (day 90) phases of stroke by neurologists blinded to the treatment allocation (i.e. a double-blind study). Moderate neurological impairment (i.e. neurological sequelae that requires partial support in daily activities) was defined as a score of ≥ 8 on NIHSS, a modified criteria reported previously [24,26]. In addition to NIHSS, assessments during admission included functional measures, Barthel Index [27], and modified Rankin Scale score [28].

### Imaging Studies

After admission, other imaging examinations included chest X-ray, routine brain computed tomography, duplex scanning of the carotid arteries, and routine cardiac analysis by 12-lead electrocardiogram and echocardiography.

The criteria for radiological diagnosis of acute IS included brain computed tomography showing a new finding of low attenuation density in focal or diffuse brain area, or MRI examination showing area(s) of high intensity (bright spots) on diffusion weighted image (DWI) MRI as described in details in our recent report [22].

### Blood Sampling and Assessment

Blood samples were obtained at 48 h (acute phase) and on day 21 (convalescent phase) after IS at 9.00 a.m. for assessment of the serum level of hs-CRP in IS patients. Blood samples were also obtained once in control subjects who participated in a health screening program in our Health Clinic at 9.00 a.m.

Venous blood was obtained from the antecubital vein into tubes with EDTA. White blood cell (WBC) count, red blood cell (RBC) count, hemoglobulin level, and biochemical data were acquired at 48 h and on day 21 after acute IS using standard laboratory methods. After centrifugation, aliquots of the serum samples were stored at -80^0 ^C before hs-CRP assay.

Serum hs-CRP level was measured by duplicated determination with a commercially available ELISA kit (R & D R&D Systems, Inc. Minneapolis, USA). The analytical range extended from 5 to 5000 pg/mL. The intra-individual variability of hs-CRP levels was assessed in both study patients and control subjects. The mean intra-assay coefficients of variance were all less than 4.0%.

### Medications

Aspirin was the first choice for patients with acute stroke in the absence of allergy, intolerance, or a history of peptic ulcer or upper gastrointestinal tract bleeding during aspirin therapy. Clopidogrel was used in patients intolerant to aspirin therapy. Other commonly used drugs included statins, angiotensin converting enzyme inhibitors (ACEIs)/angiotensin II type I receptor blockers (ARB), diuretics, calcium channel blocking agents, and beta blockers.

### Statistical Analysis

Categorical data were analyzed by Chi-square test or Fischer's exact test between two groups. Continuous variables between two groups were performed using Student *t *test. Continuous variables among the three groups were analyzed by one-way ANOVA followed by multiple comparison procedure by Bonferroni correction. The Spearman's Rank test was used to assess the correlations between quantitative variables without normal distribution. The receiver operating characteristics (ROC) curve analysis was utilized to determine which serum hs-CRP level at 48 h after acute IS was the most powerful predictive of 90-day MANE. Multivariate logistic regression analysis was utilized for identifying the independent predictors of prognostic outcomes. Statistical analysis was performed using SPSS statistical software for Windows version 13 (SPSS, Inc., Chicago, Ill., USA). A value of p < 0.05 was considered statistically significant.

## Results

### Baseline Characteristics of Study Patients and Normal Control Subjects (Table 1)

Table 1 summarizes the baseline characteristics of group 1 (EPO group) and group 2 (placebo group) patients as well as healthy control subjects. No significant difference was noted among the three groups in terms of age, gender, and body mass index. Likewise, there was no notable difference between group 1 and group 2 in coronary artery disease risk factors and the incidence of previous and old myocardial infarction. Moreover, laboratory findings showed that the RBC count, hemoglobin concentration, hematocrit, serum levels of total cholesterol, low-density lipoprotein (LDL) and creatinine did not differ among the three groups. On the other hand, serum level of hs-CRP (at 48 h) and WBC count were significantly higher, whereas the level of high-density lipoprotein was notably lower in groups 1 and 2 than those in healthy controls. No significant difference in these parameters, however, was evident between groups 1 and 2. Furthermore, both systolic and diastolic blood pressures were remarkably higher in groups 1 and 2 than in healthy control subjects, but they were similar between groups 1 and 2. Also, HbA_1C _level, incidence of extra-cranial carotid artery stenosis, and medication use did not differ between group 1 and group 2.

**Table 1:** Comparison of Baseline Characteristics and Laboratory Findings among Three Groups

Variables	Group 1 (n = 80)†	Group 2 (n = 80)†	Healthy Control (n = 60)	P value*
Age (y) (mean ± SD),	63.7 ± 11.2	66.7 ± 11.2	64.1 ± 6.0	0.134
Male, % (n)	65.0% (52)	67.5% (54)	65.0% (39)	0.932
Hypertension, % (n)	65% (52)	75% (60)	-	0.168
Diabetes mellitus, % (n)	38.8% (31)	32.5% (26)	-	0.409
Current smoking, % (n)	37.5% (30)	27.5% (22)	-	0.177
Previous stroke by history, % (n)	25% (20)	21.3% (17)	-	0.708
Previous stroke by MRI, % (n)	62.5% (50)	57.5% (46)	-	0.628
Old myocardial infarction, % (n)	8.8% (7)	6.3% (5)	-	0.781
RBC count (x10^6^/uL)	4.75 ± 0.68	4.71 ± 0.65	4.81 ± 0.64	0.561
Hemoglobin (g/dL)	14.0 ± 2.0	14.2 ± 1.7	14.1 ± 1.6	0.963
Hematocrit (%)	41.3 ± 5.8	41.4 ± 6.1	40.9 ± 6.1	0.877
WBC count (x10^3^/uL)	7.88 ± 2.41^a^	7.83 ± 2.31^a^	5.91 ± 1.84^b^	< 0.001
Total cholesterol level (mg/dL)	185.4 ± 41.0	189.0 ± 39.5	193.3 ± 36.4	0.504
HDL (mg/dL)	44.6 ± 10.9^a^	49.2 ± 17.7^a^	53.8 ± 14.8^b^	0.001
LDL (mg/dL)	115.2 ± 35.8	113.8 ± 36.1	117.2 ± 30.9	0.853
Creatinine (mg/dL)	1.01 ± 0.39	1.02 ± 0.43	1.01 ± 0.24	0.914
HS-CRP at 48 h	5.11 ± 8.79^a^	4.87 ± 7.24^a^	0.99 ± 1.11^b^	< 0.001
BMI (kg/m^2^)	25.2 ± 3.5	24.3 ± 3.9	24.7 ± 3.1	0.254
HbA_1C _level, %	6.90 ± 1.84	6.73 ± 1.85	-	0.565
SBP (mm Hg)	144 ± 20^a^	143 ± 21^a^	136 ± 18^b^	0.024
DBP (mm Hg)	85 ± 12^a^	83 ± 11^a^	80 ± 11^b^	0.041
Significant ECCA stenosis, % (n)	25% (20)	17.5% (14)	-	0.246
Statin therapy	42.5% (34)	45% (36)	-	0.750
ACEI/ARB therapy	41.3% (33)	38.8% (31)	-	0.747
EPO therapy-related adverse events				
Allergy	0% (0)	-	-	-
Polycythemia	0% (0)	-	-	-
Thrombosis event	0% (0)	-	-	-
HS-CRP at day 21	2.95 ± 4.35	2.78 ± 2.95	-	0.991

*: by t-test or Chi-square test (between the two groups) or repeated measure of NOVA (among the three groups).

Letters (^a, b^) indicate significant difference (at 0.05 level) by Bonferroni multiple comparison procedure. Same letter (^a, a^) in two groups indicate no significant difference (p > 0.05 level).

†: group 1 = with EPO treatment; group 2 = without EPO treatment.

Data are expressed as mean ± SD or % (No.) of patients.

ACEI/ARB = angiotensin converting enzyme inhibitor/angiotensin II type I receptor blocker; BMI = body mass index; DBP = diastolic blood pressure; ECCA = extra-cranial carotid artery; EPC = endothelial progenitor cell; EPO = erythropoietin; HbA_1C _= hemoglobin A_1C_; HDL = high-density lipoprotein; LDL = low-density lipoprotein; MRI = magnetic resonance imaging; SBP = systolic blood pressure; RBC = red blood pressure; WBC = white blood cell.

By day 21, the serum level of hs-CRP also did not differ between EPO group and placebo group. However, the hs-CRP level remained significantly higher in both EPO group and placebo group compared with that in the healthy control group (2.95 ± 4.35 vs. 2.78 ± 2.95 vs. 0.99 ± 1.11, respectively, all p < 0.005).

### Comparison of Neurological Status and Clinical Outcome between Patients with and without EPO Treatment (Table 2)

**Table 2:** Comparisons of Neurological Status and Clinical Outcome between IS Patients with and without EPO Treatment

Variables	Group 1† (n = 80)	Group 2† (n = 80)	P value
NIHSS at 48 h	6.69 ± 4.58	7.30 ± 7.58	0.537
Modified Rankin Scale score at 48 h	3.61 ± 1.42	2.83 ± 1.63	0.109
Barthel Index at 48 h	55.9 ± 31.0	60.6 ± 36.2	0.378
NIHSS on day 90	4.26 ± 5.41	5.53 ± 7.91	0.240
Recurrent stroke, % (n)	0% (0)	8.8% (7)	0.007
90-day mortality, % (n)	2.5% (2)	1.3% (1)	0.560
NIHSS ≥ 8.0	13.8% (11)	28.8% (23)	0.034
Combined MANE, % (n)	16.3% (13)	36.3% (29)	0.007

†: group 1 = with EPO treatment; group 2 = without EPO treatment.

IS = ischemic stroke; MANE = major adverse neurological event (defined as NIHSS ≥ 8, recurrent stroke or death on day 90 after acute IS); NIHSS = national institutes of health stroke scale.

There were no significant differences in terms of NIHSS, Barthel Index, and modified Rankin Scale score at 48 h after acute IS between patients in group 1 and group 2. Besides, the 90-day NIHSS was also similar between the two groups. Similarly, the 90-day mortality did not differ between the two groups. However, the incidence of recurrent stroke and 90-day NIHSSS ≥ 8 were remarkably higher in group 2 than in group 1. Moreover, the combined major adverse neurological event (MANE) (defined as NIHSS ≥ 8, recurrent stroke or death on day 90 after acute IS) was notably higher in group 2 than in group 1.

### Univariate and Multivariate Analysis of Predictor for 90-Day MANE (Tables 3 and 4)

All the variables in Table 1 were utilized for univariate analysis (Table 3) and the results demonstrated that hs-CRP level at 48 h was the strongest predictor of 90-day MANE. Additionally, serum levels of total cholesterol and LDL were significantly associated with 90-day MANE. Furthermore, serum level of hs-CRP at day 21 was also strongly correlated with 90-day MANE. Conversely, EPO therapy was strongly and inversely predictive of 90-day MANE. Finally, both systolic and diastolic blood pressures were significantly related to 90-day MANE. Further analysis revealed that SBP ≥ 135 mm Hg and ≤ 150 mm Hg were significantly associated with a favorable 90-day clinical outcome (p < 0.05).

**Table 3:** Univariate Analysis of Predictors for Combined MANE on Day 90 after Ischemic Stroke

Variables	Odds Ratio	95% CI	p value
Systolic blood pressure	0.981	0.963 - 0.999	0.047
Diastolic blood pressure	0.965	0.934 - 0.998	0.035
Total cholesterol level	1.011	1.022-1.02	0.015
Low-density lipoprotein	1.011	1.001 - 1.022	0.03
EPO therapy	0.341	0.161 - 0.72	0.005
HS-CRP at 48 h	1.086	1.036 - 1.140	0.001
HS-CRP at day 21	1.138	1.031-1.256	0.01

CI = confidence interval; EPO = Erythropoietin; MANE = major adverse neurological event.

**Table 4:** Multiple Stepwise Logistic Regression Analysis of Predictors for Combined MANE on Day 90 after Ischemic Stroke

Variables	Odds Ratio	95% CI	p value
Low-density lipoprotein	1.022	1.008 - 1.036	0.001
Systolic blood pressure	0.969	0.946 - 0.993	0.012
HS-CRP at 48 h	1.069	1.011 - 1.131	0.018
HS-CRP at day 21	1.163	1.021-1.324	0.023
EPO therapy	0.334	0.153 - 0.730	0.006

CI = confidence interval; EPO = erythropoietin; MANE = major adverse neurological event.

The statistically significant predictors which were shown in Table 3 were further utilized for the multiple stepwise logistic regression analysis and the results revealed that the serum level of hs-CRP level at both 48 h and day 21 after acute IS were significantly and independently predictive of 90-day MANE. In addition, the serum level of LDL was strongly and independently predictive of 90-day MANE. On the other hand, EPO therapy and systolic blood pressure were significantly and independently predictive of freedom from 90-day MANE.

### Correlation between Serum Level of hs-CRP and Neurological Status (Figures 1, 2, 3 and 4)

**Figure 1:**
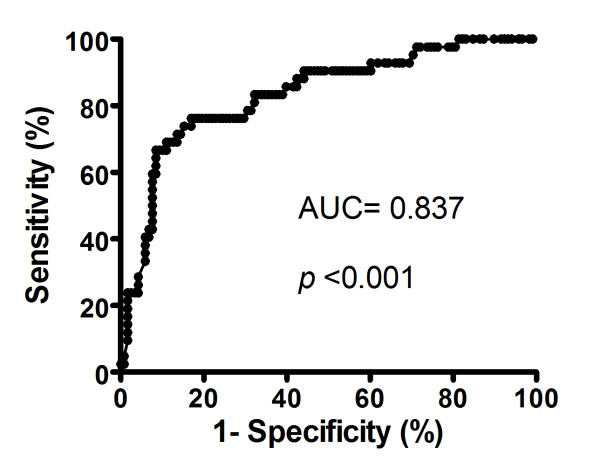
**Receiver operating characteristics (ROC) curve analysis revealed that the serum level of CRP ≥ 2.985 mg/L at 48 h after acute IS was the most powerful predictor of 90-day MANE with a sensitivity of 76.8%, a specificity of 82.7%, p < 0.001**.

The ROC curve analysis (Figure 1) revealed that serum level of hs-CRP ≥ 2.985 mg/L at 48 h after acute IS was the most powerful predictor of 90-day MANE with a sensitivity of 76.8%, a specificity of 82.7%. The positive predictive value was 61.4% and the negative predictive value was 90.5%.

**Figure 2:**
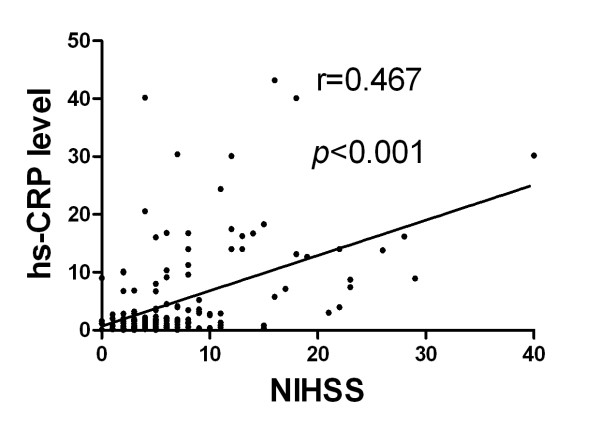
**Spearman's rank test for the correlation between serum level of high-sensitivity C-reactive protein (hs-CRP) and National Institutes Health Stroke Scale (NIHSS) at 48 h after acute ischemic stroke (p < 0.001; r = 0.467)**.

**Figure 3:**
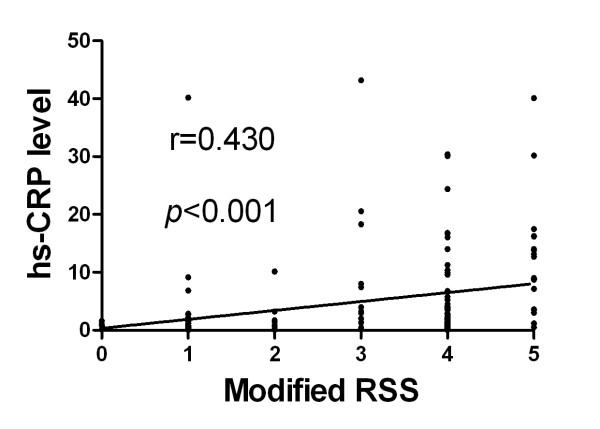
**Spearman's rank test for the correlation between serum level of hs-CRP and modified Ranking Stroke Scale (RSS) at 48 h after acute ischemic stroke (p < 0.001; r = 0.430)**.

**Figure 4:**
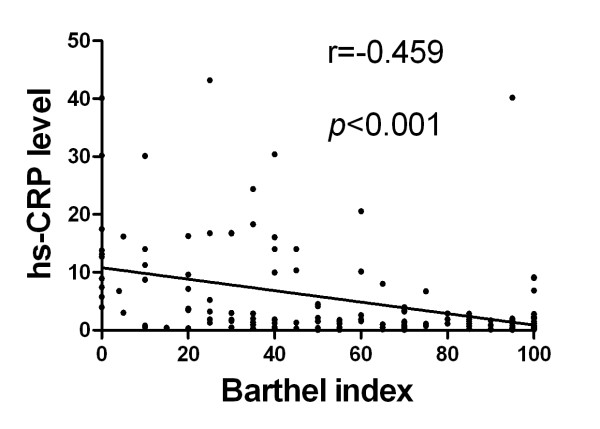
**Spearman's rank test for the correlation between serum level of hs-CRP and Barthel index at 48 h after acute ischemic stroke (p < 0.001; r = -0.459)**.

**Figure 5:**
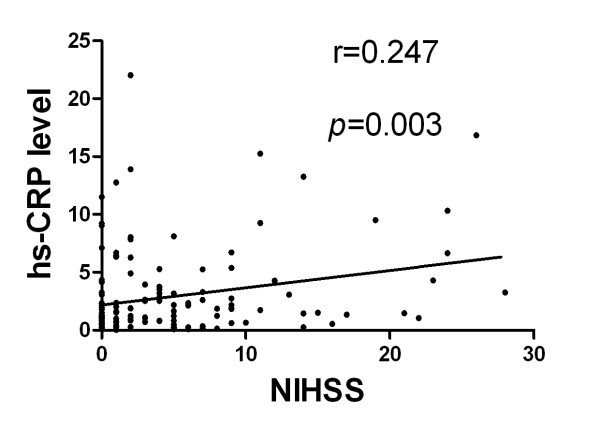
**Spearman's rank test for the correlation between 21 -day serum level of high-sensitivity C-reactive protein (hs-CRP) and National Institutes Health Stroke Scale (NIHSS) at 90-day after acute ischemic stroke (p = 0.003; r = 0.247)**.

Assessment of the correlation between hs-CRP level and degree of neurological impairment demonstrated good correlations between serum level of hs-CRP and NIHSS, Barthel Index, as well as modified Rankin Scale score at 48 h after acute IS (Figure 2, 3 &4). Besides, there was a fairly good correlation between 21-day serum level of hs-CRP and 90-day NIHSS (Figure 5).

## Discussion

This study, which compared the role of serum hs-CRP level during acute phase (at 48 hour) with that during convalescent phase (day 21) in predicting 90-day clinical outcome of patients after acute IS with or without EPO therapy, yielded several striking implications. First, hs-CRP levels during both acute and convalescent phases were remarkably higher in IS patients than in healthy control subjects. Second, EPO therapy did not significantly alter the serum level of hs-CRP. Third, hs-CRP levels during both acute phase and convalescent phase of IS were significantly and independently predictive of 90-day MANE. In contrast, EPO therapy was significantly and independently predictive of freedom from 90-day MANE.

### Inflammatory Reaction in Patients after Acute Ischemic Stroke

Consistent with the results of other recent studies [13,14], our findings also demonstrated hs-CRP level, a new pro-inflammatory index, were substantially increased in patients with IS upon admission. This finding implicated that patients with acute IS are in an inflammatory situation. Of importance is that the hs-CRP level on day 21 after acute IS, a time point when this biomarker is supposed to return to its baseline level, was found to be still significantly higher in the study patients than in healthy control subjects. This finding suggests that chronic and persistent inflammation may occur in patients even prior to acute IS. Previous studies have already shown that an elevated serum CRP level reflects an increased tendency for plaque rupture and a high atherosclerotic burden [4,6,7,29]. Additionally, oxidized LDL [30], which has been shown to be one of the strongest contributors to endothelial dysfunction and free radical generation, was found be an independent predictor of 90-MANE. The results of the present study and those from others [4,6,7,29,30], therefore, support the proposal that acute IS may be resulted from acute plaque rupture as a consequence of chronic inflammatory process.

### Level and Value of Serum hs-CRP in Patients after Acute Ischemic Stroke

The association between increased circulating level of hs-CRP and unfavorable short-term [14,15] and long-term [31-33] outcomes have been previously extensively investigated. Additionally, one [34] of these previous studies [14,15,31-33] has evaluated the serial changes of hs-CRP at acute phase (i.e. upon presentation and at 24 h and 48 h after symptom onset) of IS and showed that the circulating level of hs-CRP at 48 h after acute IS was the most predictor of unfavorable long-term clinical outcome. In the present study we also found that circulating level of hs-CRP at 48 h was better than the level of this biomarker at day 21 after acute IS for predictive of 90-day MANE. Therefore, our finding reinforced the finding of previous study [34].

Interestingly, although the level of hs-CRP during acute phase of IS has been shown to be significantly predictive of the severity of stoke and prognostic outcome [14,15,31,32], the value of hs-CRP level at convalescent stage of IS in predicting clinical outcome has not been addressed. The novel finding in the present was that circulating level of hs-CRP at day 21 after acute IS remained significantly and independently predictive of 90-day MANE. Therefore, our finding extended the finding of previous studies [14,15,31-35].

The importance of the current study is that hs-CRP levels were obtained from the same group of study patients during both acute and convalescent phases for determining the association of this biomarker with clinical outcome. In addition, another important finding is that hs-CRP level at 48 h after IS was significantly and strongly correlated with NIHSS, Barthel Index, and modified Rankin Scale score. Above all, the most important finding is that hs-CRP levels during both acute and convalescent phases were significantly and independently predictive of 90-day MANE. Our findings, therefore, support the validity of using serum hs-CRP level in predicting clinical outcome in patients with and without IS regardless of the timing for blood sampling [9,11,15,15,31-33].

### Impact of EPO Therapy on Serum hs-CRP Level and 90-day MANE

Some clinical observational studies [36,37] have shown that EPO therapy improved the clinical outcome of patients after acute IS. The essential finding in the present study is that EPO therapy was an independent predictor of improvement in 90-day MANE. Therefore, our findings, in addition to strengthening those of previous studies [36,37], highlight the therapeutic potential of EPO in patients after IS who are not suitable candidates for thrombolytic therapy.

The baseline variables (Table 1) were identical in group 1 and group 2 patients upon presentation, therefore, it is not surprising that the circulating level of hs-CRP did not differ between group 1 and 2 at 48 hour after acute IS. Surprisingly, while EPO has been reported to be anti-inflammatory [19,20,38] and hs-CRP has been widely accepted as an important inflammatory biomarker for predicting prognostic outcome of cardiovascular and cerebrovascular diseases [4,6,7,9-11,14,15,31,32], serum hs-CRP levels at day 21 after IS did not differ between the patients with and those without EPO therapy. However, the results of the present study identified EPO therapy as an independent predictor of freedom from 90-MANE. This finding supports the mechanisms underlying the improvement in clinical outcome after EPO therapy against acute IS through enhancing circulating level of EPCs [24] rather than via inhibiting inflammatory response.

### Blood Pressure upon Admission Independently Predictive of 90-Day MANE

The association between blood pressure and clinical outcome after acute IS has been extensively investigated in previous studies [39-41]. Control of systolic blood pressure to an optimal level around 140-150 mm Hg has been shown to improve clinical outcome after acute IS [40]. In the present study, the mean systolic blood pressure of our patients was 140 mmHg. Further analysis revealed that SBP ≥ 135 mm Hg and ≤ 150 mm Hg was significantly associated with a favorable 90-day clinical outcome. Also of importance in the current study is that systolic blood pressure was found to be independently predictive of freedom from 90-day MANE. Therefore, our findings were consistent with those of previous studies [39-41].

### Study Limitation

First, this study only chose two time points rather than constant blood samplings during the whole time course to determine the changes in serum level of hs-CRP in patients after acute IS. Therefore, we did not provide information on the serial changes in circulating hs-CRP level after IS. Second, although this study did not show significant reduction in serum hs-CRP level following a standard EPO dosage, whether a higher dose of EPO would actually reduce the serum level of hs-CRP in the setting of acute IS remains unclear.

## Conclusion

Serum level of hs-CRP during both acute and convalescent phases was significantly and independently predictive of 90-day MANE, whereas EPO therapy was significantly and independently predictive of freedom from 90-day MANE. However, EPO treatment did not diminish the predictive value of hs-CRP for unfavorable clinical outcome after acute IS.

## Competing interests

The authors declare that they have no competing interests.

## Authors' contributions

All authors have read and approved the final manuscript.

YKH, TTH, CHT, and SL designed the experiment, drafted and performed animal experiments. CSY, SS, CYL, LHS and YCM were responsible for the laboratory assay and troubleshooting. YKH, THT and HKY participated in refinement of experiment protocol and coordination and helped in drafting the manuscript.

All authors report no disclosures and have any commercial associations or interests, including consultancies, stock ownership or other competing equity interest.

Tzu-Hsien Tsai contributed equally as the first author to this work. Chun-Man Yuen contributed equally compared with the corresponding author to this work.
